# Driving forces of herbaceous species diversity in natural broadleaf forests from in Maoershan from Northeast China

**DOI:** 10.3389/fpls.2024.1449421

**Published:** 2024-08-22

**Authors:** Qi Sheng, Lingbo Dong, Zhaogang Liu

**Affiliations:** Key Laboratory of Sustainable Forest Ecosystem Management-Ministry of Education, College of Forestry, Northeast Forestry University, Harbin, China

**Keywords:** stand structure, herbaceous species diversity, soil nutrient, structural equation model, broadleaf forests

## Abstract

The understory herbaceous flora plays a pivotal role in regulating the structural stability, complexity, and ecological function of forest communities. It is crucial to investigate the impact of the intricate connections between these factors and the forces driving the diversity of herbaceous species within natural broadleaf understory forests can assist forest managers in developing optimal forest structure optimization techniques, allowing them to adjust the forest species diversity. In this study, Pearson correlation analysis, conventional correlation analysis, and multiple linear regression were employed to elucidate the relationship between stand structure, soil nutrients, and understory herbaceous species richness in natural broadleaved forests. Structural equation modeling was utilized to ascertain the influence of multiple factors on understory herbaceous species diversity and to evaluate the underlying pathways. The results indicated a significant negative correlation between stand closure and the Simpson’s and Shannon-Wiener’s indices, and between the mixing degree and the Pielou evenness index, Simpson’s index, and Shannon-Wiener’s index (p<0.05). Furthermore, a significant positive correlation was observed between soil nutrients, specifically organic matter and total phosphorus, and the Pielou evenness index and Shannon-Wiener’s index (p<0.05). It was found that total phosphorus was significantly positively correlated with both the Pielou evenness index and the Shannon-Wiener index (p<0.05). The correlation coefficients of the first group of typical variables in the typical correlation analysis were 0.498 and 0.585, respectively (p<0.05). From the set of typical variables of stand structure, it can be seen that the Hegyi competition index and the canopy density affected the diversity of understory herbaceous plants. The composite index demonstrated the greatest impact, with loadings of 0.872 and -0.506, respectively. The Simpson and Shannon-Wiener indices exhibited the most sensitive loadings of -0.441 and -0.408, respectively. The soil nutrients of SOM and TN affected the understory herbaceous plant species diversity composite index, with greater loadings of -0.184 and 1.002, respectively. The path coefficient of the understory herbaceous diversity stand structure was 0.35. The path coefficient with soil nutrient content was found to be 0.23 following structural equation analysis and the path coefficient between stand structure and soil nutrient content was 0.21, which indirectly affect the diversity of understory herbaceous species. To enhance the diversity of herbaceous species, it is recommended that the canopy density and tree density of the upper forest be reduced appropriately, while the degree of mixing and the level of spatial distribution of trees be adjusted in a manner that maintains a reasonable stand structure. Furthermore, a comprehensive forest management program for improving soil nutrients should be considered.

## 1 Introduction

Forest ecosystems play an irreplaceable role in maintaining ecological balance and promoting the development of human society as the mainstay of China’s terrestrial ecosystems (Liang and Wei, 2020). Understory vegetation and forest soils are important components of forest ecosystems, and understory species diversity is one of the most important indicators of ecosystem stability, and an increase in understory species diversity is conducive to improving soil nutrient content and water holding capacity, etc (Aubert et al., 2004; Ampoorter et al., 2015). Understory vegetation and forest soils are important components of forest ecosystems, and understory species diversity is one of the most important indicators of ecosystem stability (Liu et al., 2016). Forest soil nutrient content is an indicator of soil fertility, providing plants with the nutrients they need to grow and survive (Laughlin et al., 2007). As forest structure is the most likely factor to be regulated in the process of forest management, it is of great importance to investigate the relationship between forest spatial structure, understory species diversity, and soil nutrient content for the sustainable management of forest resources (Barbier et al., 2008; Tatsumi, 2020).

In recent years, many studies have been carried out by national and international researchers on the relationship between species diversity and soil nutrient levels (Uddin and Robinson, 2017; Wu et al., 2024). The relationship between vegetation and soil nutrient content in restored understory was investigated and the results showed that the diversity index of restored understory shrubs and grasses was most influenced by soil total nitrogen, total phosphorus, total potassium, quick potassium organic matter, and water content (Zemunik et al., 2018; Su et al., 2022).

The correlation analysis between the understory vegetation and the physio-chemical properties of the soil indicates that the nitrogen, phosphorus, and potassium content of the soil, as well as its pH value, are key factors that determine the species diversity of the understory vegetation. Many studies have demonstrated the close relationship between understory vegetation and soil nutrient content (Mao et al., 2021). The forest spatial structure is an important factor that influences the species diversity of the understory vegetation and also has a significant effect on the nutrient content of the soil (Cao et al., 2020). The degree of mixing and the distribution pattern of forest trees are important factors that influence the diversity of shrub species in the understory (Yang et al., 2023; Alaina et al., 2000). The degree of mixing and the forest layer index also have a significant effect on the nutrient content of the soil. They are the main factors that influence the content of soil organic matter, total nitrogen, and active phosphorus (Mestre et al., 2017; Cui et al., 2022).

Structural equation modeling (SEM) is an extension of general linear modeling (GLM) that combines factor analysis with systems of equations to validate interrelationships between variables (Libório et al., 2020). To investigate the relationship between stand structure, soil nutrient content, and understory herbaceous species diversity in natural broadleaf forests, this paper selects 152 plots of broadleaf forests in Maoershan Forest Farm (1) Pearson’s correlation analysis and typical correlation analysis are mainly used to analyses the effects of stand structure and soil nutrient content on the diversity of understory herbaceous species, (2) multiple regression and structural equation modeling (SEM) are then used to further investigate the correlation between the spatial structure factor of the forest stand, the diversity of understory herbaceous species and the soil nutrient content factor The majority of forest types are natural secondary broadleaf mixed forests in Maoershan Forest Farm, and the district forest climax community is *Pinus koraiensis Siebold* broadleaf forest. However, there is a paucity of research examining the factors influencing understory herbaceous diversity, and there is a dearth of conservation efforts directed towards forest species diversity during forest management. Consequently, it is significant ecological importance that this study to promote the restoration of top zone communities and enhance forest diversity of the natural broadleaf forests. The aim was to provide a basis for the adjustment of the stand structure of broadleaf forests to improve the stability of the natural broadleaf forests and provide a scientific basis for the sustainable management and conservation of forests.

## 2 Method

### 2.1 Study site and data collection

**Figure 1:**
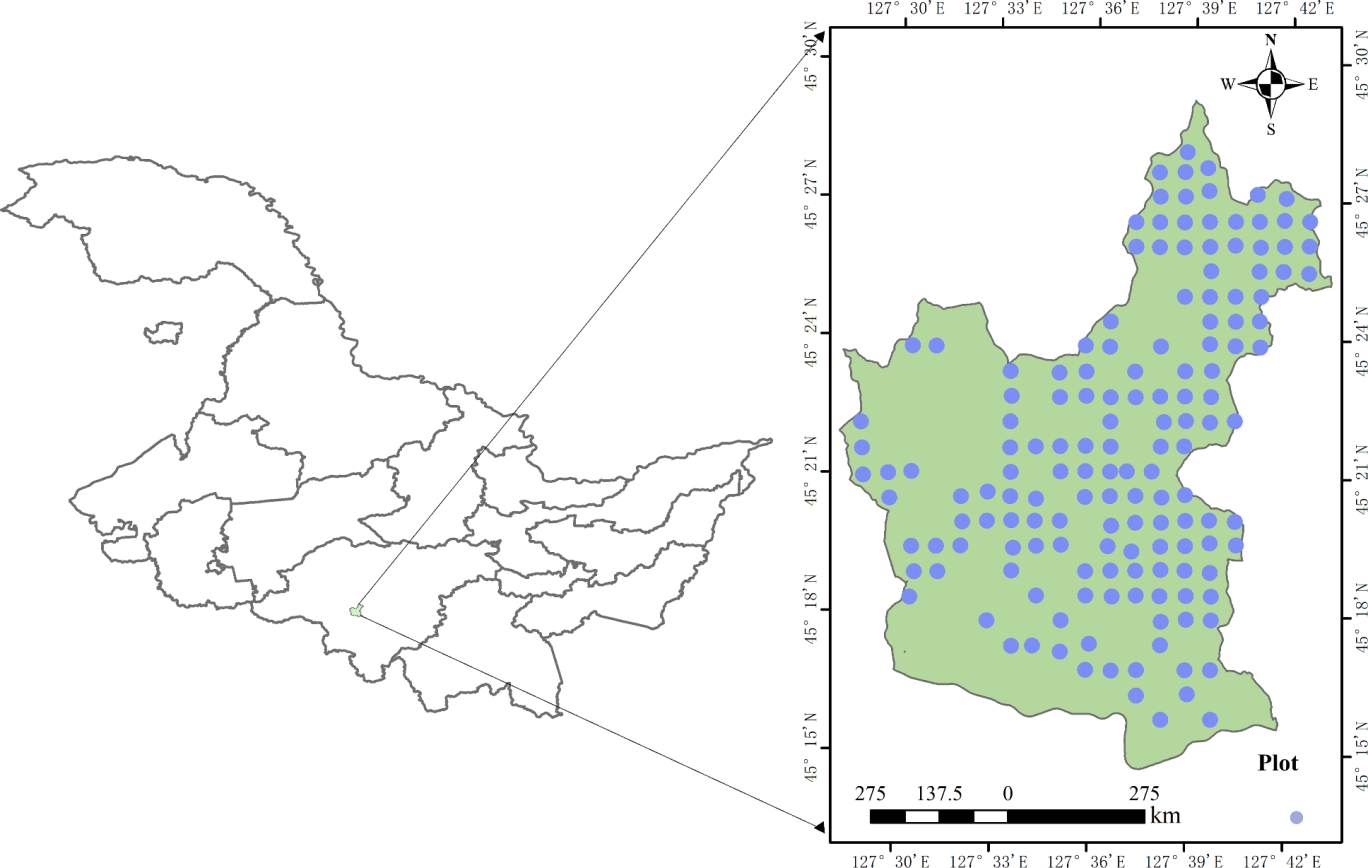
The locations of the studied forest stand in Heilongjiang Province in northeast China and the distribution of the studied plots in Maoershan Forest Farm.

The research area of this study is located in Maoershan Forest Farm in the southeast of Heilongjiang Province (45°20′–45°25′ N, 127°30′–127°34′ E), with a total area of 26,453.7 ha (**Figure 1**). The area belongs to a typical low mountainous, hilly area, with an average slope of ~10°–15°, and each slope level is evenly distributed in the forest, with an average elevation of 381 m. The area has a temperate continental monsoon climate with short summers and long winters, a mean annual temperature of 3.0°C, and a mean annual rainfall of 723.8 mm. The area has an abundance of tree species including *Pinus koraiensis Siebold & Zucc.*, *Picea asperata Mast.*, *Larix gmelinii (Rupr.) Kuzen*, *Fraxinus mandshurica Rupr.*, *Juglans mandshurica Maxim.*, *Quercus mongolica Fisch.*, *Tilia tuan Szyszyl.*, *Acer pictum Thunb.*, *Ulmus pumila L.*, *Betula platyphylla*, and *Populus davidiana.* The study area is rich in vegetation types consisting mainly of natural secondary forest stands in different stages of succession, including broadleaf mixed forests dominated by valuable species, birch forests, and oak forests. Natural secondary forests are plant communities that grow and reproduce naturally after the destruction of primary forests. Natural secondary forests are a category of forests that have formed following human or natural disturbances such as clearing, grazing, logging, hunting, and fire in natural forests.

The data presented in this paper were obtained from the second-class forest resources survey of Maoershan Forest Farm in 2016. A total of 152 plots were surveyed in the natural broad-leaved forests of Maoershan Forest Farm, Each plot was 0.06 hm which was then divided into 10 m × 10 m grids by the adjacent grid method, and all trees with DBH ≥ 5 cm at breast height were recorded for tree species, number, diameter at breast height (DBH), tree height (H), crown width, condition, and coordinates (**Table 1**). To investigate the understory herbaceous layer community, five 1 m × 1 m herbaceous sample plots were set up in each plot, and the height, cover, abundance, and density of each species were recorded in the herbaceous plots. A five-point sampling method was employed to obtain mixed soil samples from the 0-20 cm depth interval at each plot, after which the samples were transported to the laboratory for further analysis for the determination of soil pH, organic matter, total nitrogen, total phosphorus, alkaline dissolved nitrogen, effective phosphorus, and quick-acting potassium.

**Table 1:** Basic characteristics of the studied plots.

Plot	Height of dominant tree(m)	Mean DBH (cm)	Mean Elevation (m)	Canopy Density
1	21.7	18.4	598	0.8
2	21.2	22.4	610	0.7
3	20.1	27.5	527	0.6
4	20.3	26.5	580	0.7
5	17.8	26.0	530	0.8
…				
148	19.2	20.5	536	0.7
149	21.3	21.9	524	0.8
150	25.0	14.0	497	0.8
151	20.0	19.0	600	0.9
152	19.7	20.5	541	0.7

### 2.2 Variable selection and data processing

Stand structure is mainly divided into stand spatial structure and stand non-spatial structure parameters. In this study, in terms of stand non-spatial structure parameters, the following four indices were adopted: tree height, DBH, canopy density, and tree density, and in terms of stand spatial structure parameters, the following four indices were adopted: complete mingling index, uniform angle, dominance and Hegyi competition index (Hui et al., 1999, 2011; Hui et al., 2004a; Hui et al., 2004b).

The stand spatial structure is based on the stand spatial structure unit. Each tree in the forest and its adjacent trees (n) can form a stand-spatial structure unit. To avoid the adjacent trees of the object tree on the boundary falling outside the sample plots and thus affecting the results, a 5 m buffer zone was set, the tree in the buffer zone could not be set as the object tree, and the number of adjacent trees n was 4 (Bettinger and Tang, 2015) (**Table 2**).

**Table 2:** Indicator definition and formula.

Variable		Formula	Define
Spatial structure	Complete mingling index (*M_c_*)	$M_{c_{i}}=\frac{1}{2}\left(D_{i}+\frac{C_{i}}{n_{i}}\right)\cdot M_{i}$	*where* $C_{i}/n_{i}$ *is isolation for nearest neighbor tree species;* $C_{i}$ *is the number of different species in adjacent pairs of all neighboring trees;* $n_{i}$ *is the number of the nearest neighboring trees;* $D_{i}$ *is the Simpson index of the spatial structure unit i*, $D_{i}=1-\sum_{j=1}^{S_{i}}p_{j}^{2};$ $S_{i}$ *is tree species of the spatial structure unit I;* $p_{j}\;$ *is the proportion of trees of the jth species;* $M_{i}$ *is a simple mingling index*, $M_{i}=\frac{1}{n}\sum_{j=1}^{n}v_{ij}$ , $v_{ij}=1$ *, if reference tree i and its neighbor tree j are of different tree species, otherwise*, $v_{ij}=0$
	Uniform angle index (*W*)	$W_{i}=\frac{1}{n}\sum_{j=1}^{n}w_{ij}$	*where α is the angle of two neighbor trees of the spatial structure unit if the angle α of two neighbor trees;* $where:w_{ij}=\left\{\begin{array}{c} 1;if\;\alpha_{j}<\alpha_{0}=72^{{}^{\circ}}\\ 0;otherwise \end{array}\right\}$
	Dominance index (*U*)	$U_{i}=\frac{1}{n}\sum_{j=1}^{n}K_{ij}$	*where z_ij_ takes the value 1 if the jth neighbor(dj) is smaller than the reference tree i(d_i_), and the value 0, otherwise*, $z_{ij}=\left\{\begin{array}{c} 1;d_{j}<d_{i} \end{array}\right\}$
	Hegyi competition index (*CI*)	$CI_{i}=\sum_{j=1}^{n_{i}}\frac{d_{j}}{d_{i}\cdot L_{ij}}$	*where d_i_ and d_j_ represent the diameter at breast height of reference tree i and its neighbor tree j, respectively; and L_ij_ represents the Euclidean distance between reference tree i and its neighbor tree j.*
Species Diversity (Oksanen et al., 2018)	Shannon–Wiener index	$\;H=-\sum_{i=1}^{s}P_{i}lnP_{i}\;$	*where S is the number of species, N is the total number of individuals of all species, and Pi is the ratio of the number of the ith species to the overall number of species.*
	Pielou evenness index	$E=-\sum_{i=1}^{s}P_{i}lnP_{i}/lnS\;$	
	Simpson index	$D=1-\sum_{i=1}^{s}P_{i}^{2}$	

Five soil nutrient indicators, including soil organic matter (SOM), total nitrogen (TN), total phosphorus (TP), available potassium (AK), and pH were selected. The content of the SOM was determined by using the potassium dichromate oxidation method; The TN content was measured by using the Kjeldahl method (Forest soil analysis methods) (State Forestry Administration, 1999). The TP content was measured by using the sodium hydroxide alkaline solution molybdenum antimony anti-colorimetric method; the AK content was measured by using the ammonium acetate leaching-flame photometric method (Smith and Bain, 1982; Sims, 1989).

### 2.3 Data statistical analysis

We adopted the Pearson correlation analysis, typical correlation analysis, multiple linear regression, and structural equation model to explore the relationships between stand structure, understory herbaceous species diversity, and soil nutrient content with R 4.1.3.

#### 2.3.1 Pearson’s correlation analysis

The Pearson correlation coefficient (Ali et al., 2019) was used to calculate the univariate correlation between stand structure and understory herbaceous plant species diversity with the following Equation 1:


(1) $$r=\frac{\sum^{\;}\left(x-\bar{x}\right)\left(y-\bar{y}\right)}{\sum^{\;}{\left(x-\bar{x}\right)}^{2}\sum^{\;}{\left(y-\bar{y}\right)}^{2}}$$

where *r* is the correlation coefficient, *x* and *y* are samples, and *x* and *y* are the average of samples.

#### 2.3.2 Canonical correlation analysis

Canonical Correlation Analysis (CCA) (Shaheen et al., 2011) is a mathematical method used to measure the correlation between two sets of multiple variables, assuming that the two sets of variables are X(x_1_,x_2_,x_3_,…x_p_) and Y(y_1_, y_2_, y_3_,… y_q_); $\text{α}=\left(\alpha_{1},\alpha_{2}\cdots \alpha_{p}\right),\beta =\left(\beta_{1},\beta_{2}\cdots \beta_{q}\right)$ One or more representative comprehensive variables, *U* and *V* were selected among the two sets of variables, r is the correlation coefficient between *U* and *V* (Equations 2, 3).


(2) $$U=\alpha {\hat{\;}}^{T}X$$

(3) $$V=\beta {\hat{\;}}^{T}Y$$

where one set of variables is the stand structure indicators, the other is soil nutrient indicators, and the other is understory herbaceous plant species diversity indicators.

#### 2.3.3 Multiple linear regression

Stepwise regression was conducted to establish a multiple linear regression model with species diversity indices as the dependent variable; soil nutrient indicators and stand structure indices as the independent variables, and the equation was as follows (Equation 4).


(4) $$y=\beta_{0}+\beta_{1}x_{1}+\beta_{2}x_{2}+\;.\;.\;.\;+\beta_{p}x_{p}+\mu$$

where *y* is the dependent variable, *x_p_* is the independent variable, *β_p_* is a constant, and *μ* is the residual

#### 2.3.4 Structural equation model

The structural equation model (SEM) is a multivariate method of statistical analysis used for building, estimating, and testing causal models. It includes regression analysis, factor analysis, path analysis, and multivariate analysis of variance, which is divided into measurement models and structural models. The formulae are as follows (Equations 5, 6) (Su et al., 2024):


(5) $$X=\Lambda_{x}\xi +\sigma$$

(6) $$Y=\Lambda_{Y}\eta +\epsilon \;$$

(7) η=Βη+Γξ+ζ

where X is the measurement variable for *ξ*, Y is the measurement variable for *η*, *ξ* is the exogenous latent variable, ηis the endogenous latent variable, *δ ϵ*, are the measurement error vectors, and $\Lambda_{x},\Lambda_{y}$ are the correlation coefficient matrices for the measurement variables X, Y and the latent variables ξ, η. Equation 7 is a structural model that describes the latent variables causal connection, where B represents the correlation coefficient matrix among the endogenous latent variables, *Γ* represents the influence of exogenous latent variables *ξ* on endogenous latent variables *η*, and *ζ* represents the unexplained part of the model, which is the error in endogenous latent variables.

## 3 Results

### 3.1 Basic characteristics of stand structure, nutrient contents of soils, and species diversity

There were 128 herbaceous plant species in the selected sample plots, involving 47families and 79 genera, among which Asteraceae was the most abundant, accounting for 8.6% of the total number of herbaceous plant species, followed by Rosaceae and Ranunculaceae.

The basic information of stand structure parameters (stand non-spatial structure and stand spatial structure) the understory herbaceous plants species diversity index and the nutrient contents of soil plots is shown in **Table 3**, from which it can be seen that the coefficient of variation of the indices from 4% to 53.34%. Additionally, the coefficient of variation of the nutrient contents of soils was large among the plots, the largest is soil organic matter 53.34%. The variation in dominance index, uniform angle, and pH was small among the plots, and the coefficients of variation were 4.0%, 5.35%, and 6.64%, respectively. The coefficient of variation of species diversity which were29.18%, 7.95%, and 15.38%, respectively.

**Table 3:** Basic information of stand structure and species diversity index of the plots.

Category	Index	Mean ± Standard Deviation	Minimum	Maximum	Coefficient of Variation (%)
Stand structure	Tree height(m)	20.24 ± 3.65	13.00	33.00	18.03%
	DBH(cm)	19.41 ± 4.25	11.20	35.30	21.89%
	Canopy density	0.71 ± 0.13	0.6	0.9	18.30%
	Tree density(N•ha^-1^)	919 ± 346	200	2250	37.64%
	Complete mixing index	0.63 ± 0.15	0.10	0.88	23.81%
	Uniform angle index	0.56 ± 0.03	0.48	0.65	5.35%
	Dominance index	0.50 ± 0.02	0.36	0.56	4.0%
	Hegyi competition index	3.38 ± 0.96	1.51	6.07	28.40%
Species diversity	Shannon–Wiener index	1.85 ± 0.54	0.50	2.81	29.18%
	Pielou evenness index	0.88 ± 0.07	0.62	1.00	7.95%
	Simpson index	0.78 ± 0.12	0.32	0.93	15.38%
Nutrient contents of soils	SOM(g•kg^-1^)	153.90 ± 82.10	27.86	541.22	53.34%
	TN(g•kg^-1^)	9.32 ± 4.15	2.23	35.57	44.52%
	TP(g•kg^-1^)	1.31 ± 0.45	0.49	4.71	34.35%
	AK(mg•kg^-1^)	282.82 ± 128.46	58.59	880.92	45.42%
	pH	6.02 ± 0.40	4.92	7.06	6.64%

**Figure 2:**
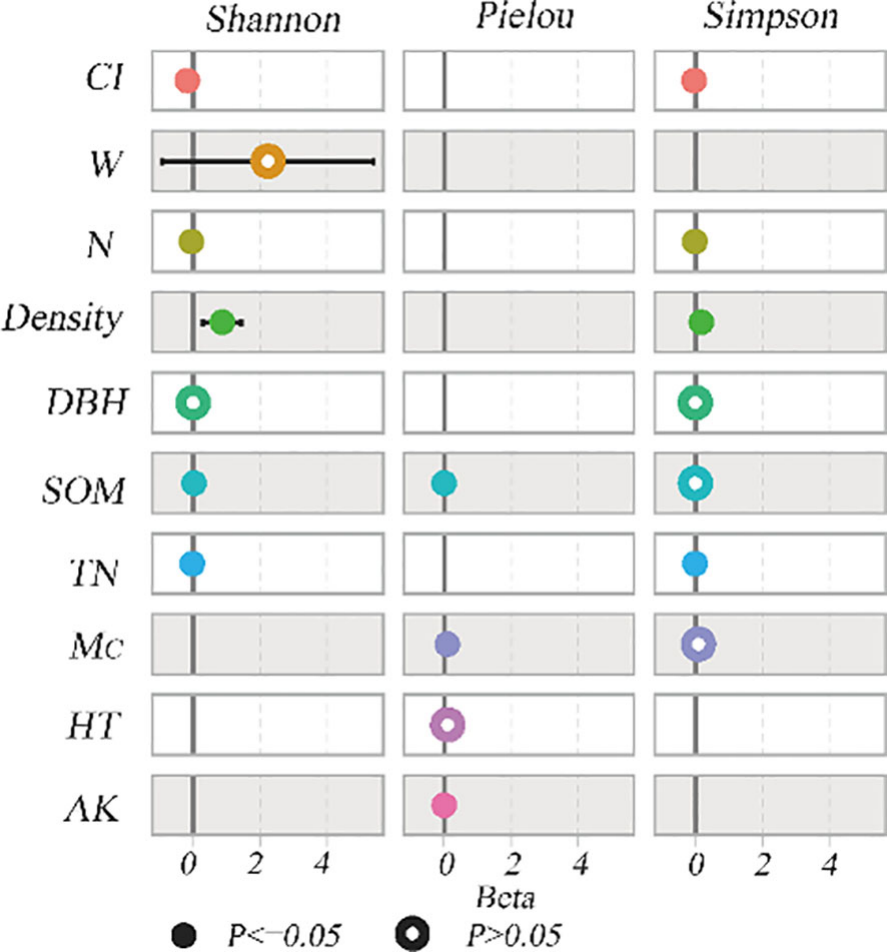
Correlation coefficients between stand structure and understory herbaceous plant species diversity. H, Tree Height; Density, Canopy density; N, Tree density; W, Uniform angle index; M_c_, Complete mixing index; CI, Hegyi competition index; SOM, soil organic matter; TN, total nitrogen; AK, available potassium.

The univariate correlation analysis between stand structure soil nutrient content and understory herbaceous plant species diversity was performed using the Pearson correlation coefficient. as shown in **Figure 2**. The result indicates that the Pielou evenness index was significantly correlated with the Complete mixing index and Uniform angle index of stand spatial structure (p< 0.05), and it was significantly correlated with soil organic matter and total phosphorus of soil nutrient content (p < 0.05), it was significantly correlated with total nitrogen and available potassium of soil nutrient content (p < 0.01). The Simpson index was significantly correlated with the Complete mixing index and Hegyi competition index of the stand spatial structure (p< 0.05), and it was significantly correlated with the Canopy density of the stand structure (p< 0.05). The Shannon–Wiener index was significantly correlated with the Complete mixing index of stand spatial structure (p< 0.05), and it was significantly correlated with Canopy density and Tree density of stand structure (p< 0.05) and it was significantly correlated with soil organic matter and total phosphorus of soil nutrient content (p<0.05), it was significantly correlated with total nitrogen and available potassium of soil nutrient content (p < 0.01). In **Figure 2**, tree height and DBH were not significantly correlated with understory herbaceous plant species diversity (p > 0.05).

### 3.2 Typical correlation analysis between stand structure and understory herbaceous plants species diversity

The five stand structure indices were considered as one set of variables (U1), the four soil nutrient content indices were considered as one set of variables (Z1), and the three species diversity indices were considered as another set of variables (V1). The linear relationship between each two sets of variables was obtained by performing a typical correlation analysis.

It is shown in **Table 4** that the typical correlation coefficient between the first group of typical variables was 0.498, and 0.585, respectively, and the results passed the typical correlation coefficient test at the α = 0.05 level, indicating that there was a strong correlation between each two groups of variables. Therefore, the group of variables consisting of five stand structure indices, and the four soil nutrient content indices could be used to explain the group of understory herbaceous plant species’ diversity variables; however, all three groups of typical variables did not pass the significance test. Therefore, only the first group of typical variables was analyzed.

**Table 4:** Canonical correlation coefficient and its significance test.

	Typical Variable Group	Canonical Correlation Coefficient	*p*
Stand structure	1	0.498	0.000
	2	0.139	0.664
	3	0.085	–
Nutrient contents of soils	1	0.585	0.041
	2	0.196	0.602
	3	0.072	0.482

The equation for the first group standardized coefficients of typical variables is shown in **Table 5**, In the linear combination U1, the loads of X3 (Hegyi competition index) and X5 (Canopy density) were larger, at 0.872 and-0.506, respectively. From the cross-loading coefficients in **Table 5**, it can be seen that the loads of X3 (Hegyi competition index) and X5 (Canopy density) were larger, at 0.312, and -0.201, respectively, indicating that Hegyi competition and Canopy density, were the dominant factors in the comprehensive index of stand non-spatial structure. In the linear combination V1, Y1(Simpson index) and Y2 (Shannon–Wiener index) had larger loads of 0.364 and -1.312, respectively, the cross-loading coefficients of Y1(Simpson index) and Y2 (Shannon–Wiener index) were larger, at -0.441, and -0.408, respectively, indicating that the Simpson index and Shannon–Wiener index played a decisive role in the comprehensive index of understory herbaceous plants species diversity.

**Table 5:** Standardization coefficient and cross-load coefficient of typical variables.

	Variable		X1	X2	X3	X4	X5	Y1	Y2	Y3
stand structure	Standardization coefficient	U1	-0.229	0.062	0.872	-0.213	-0.506			
		V1						0.364	-1.312	-0.153
	Cross-load coefficient	U1	0.100	0.103	0.312	-0.044	-0.201			
		V1						-0.408	-0.441	0.109
Nutrient contents of soils	Standardization coefficient	Z1	-0.184	1.002	0.054	0.134	–			
		V1						-1.711	0.763	0.831
	Cross-load coefficient	Z1	0.209	0.273	0.201	0.201				
		V1						-0.220	0.112	0.196

X1, Complete mixing index; X2, Uniform angle index; X3, Hegyi competition index; X4, Tree density; X5- Canopy density in U1; X1, soil organic matter; X2, total nitrogen; X3, total phosphorus; X4, available potassium in Z1; Y1, Simpson index; Y2, Shannon–Wiener index; Y3, Pielou evenness index.

Additionally, **Table 5** showed that loads of X1 (SOM), X2 (TN) were larger, at -0.184, 1.002, respectively, the cross-loading coefficients of X1 (SOM), X2 (TN) were larger, at0.209, 0.273, respectively in Z1; and loads of Y1 (Simpson index) and Y3 (Pielou evenness index) in V1 were the largest, at-1.711and 0.831, respectively, the cross-loading coefficients of Y1 (Simpson index) and Y3 (Pielou evenness index) in V1 were the largest, at -0.220 and 0.196, indicating that SOM and TN, were the dominant factors in the comprehensive index of nutrient contents of soils and the Simpson index and Pielou evenness index played a decisive role in the comprehensive index of understory herbaceous plants species diversity.

### 3.3 Multiple stepwise regression results

**Figure 3:**
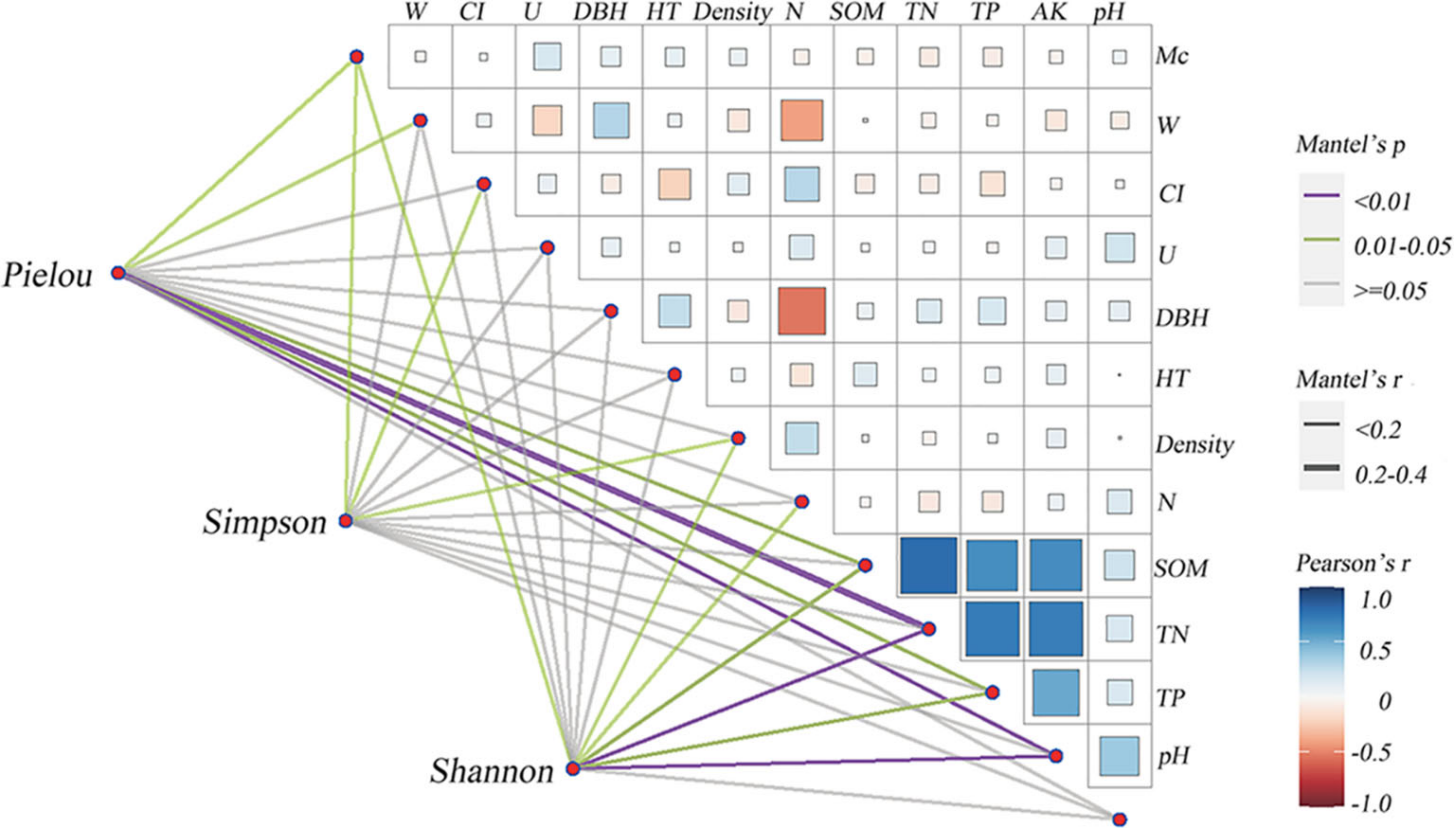
Correlation coefficients between stand structure, nutrient contents of soils, and understory herbaceous plant species diversity. HT, Tree Height; Density, Canopy density; N, Tree density; W, Uniform angle index; U, Dominance index; M_c_, Complete mixing index; CI, Hegyi competition index; SOM, soil organic matter; TN, total nitrogen; TP, total phosphorus; AK, available potassium.

Regression equations for each species diversity index were obtained using multiple regression, reflecting the stand structure and soil nutrient content indices which were significantly associated with understory herbaceous plants species diversity. As can be seen from **Figure 3**, Canopy density, *CI*, SOM, and TN had a considerable effect on the Shannon–Wiener index(p<0.05). *M*c, SOM, and AK played a key role in significantly affecting the Pielou evenness index(p<0.05). Canopy density *CI*, N, and TN had a considerable effect on the Simpson index (p<0.05).

Canopy density, *CI*, and TN were excluded from the multiple regression equations of the Pielou evenness index. *W*, Tree height, and DBH were not significant (p>0.05) from the multiple regression equations of all species’ diversity indices, indicating that the effect of DBH and tree height on the understory herbaceous plant species diversity was small.

### 3.4 Structural equation model results

**Figure 4:**
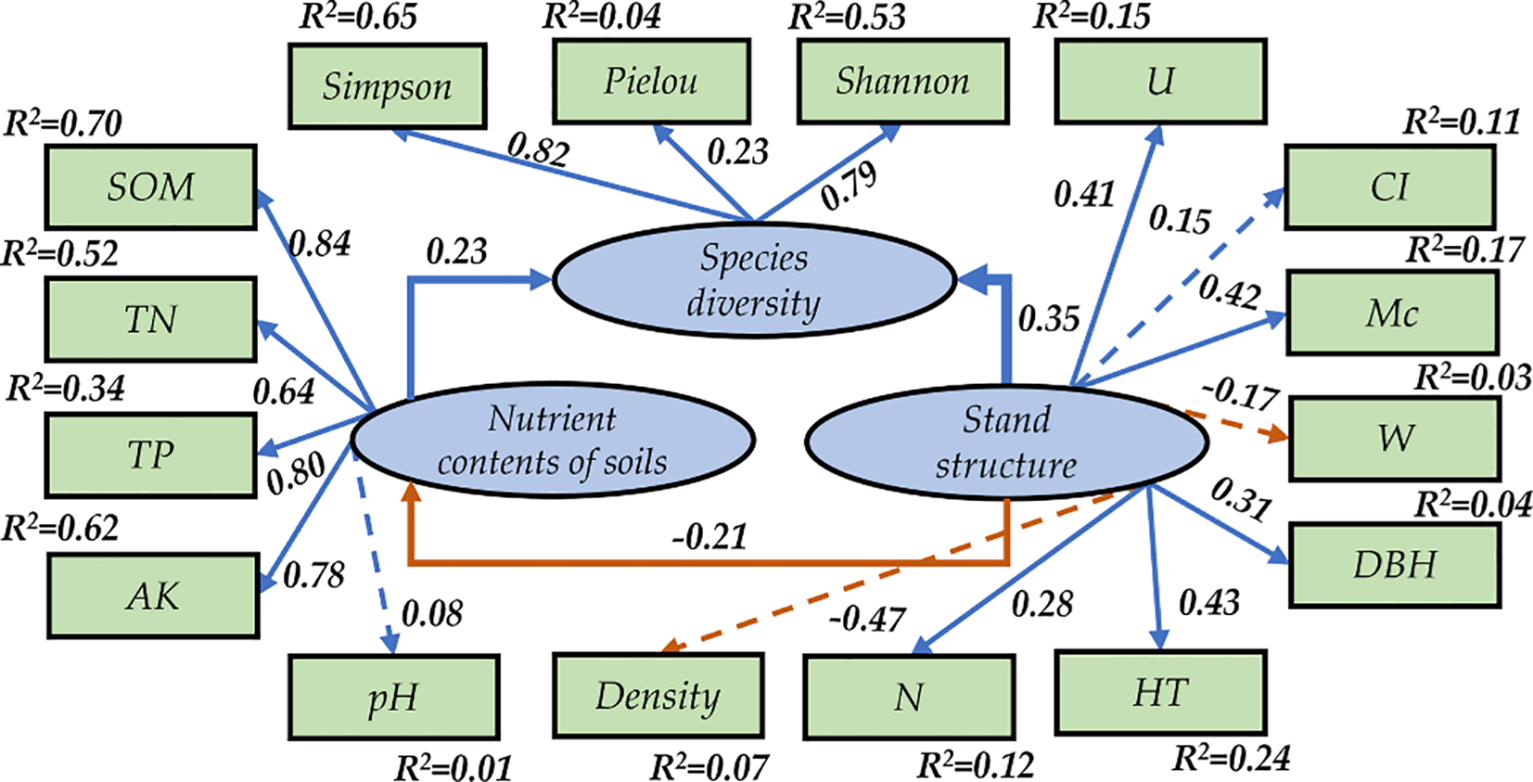
Modified model for the evaluation of soil fertility. R^2^ indicates the amount of variance explained by the model.

As shown in **Figure 4**, the path coefficients for the observed variables including *HT*, *DBH*, *N*, *Density*, *M_c_*, *W*, *CI*, and *U* to stand structure indicators were 0.43, 0.31,0.28,0.47,0.42,0.17,0.15 and 0.41 (**Figure 4**). The path coefficients for the observed variables including *SOM*, *TN*, *TP*, *AK*, and *pH* to soil nutrient contents indicators were 0.84, 0.64,0.80, 0.78, and 0.08 (**Figure 4**), respectively. The path coefficients of the observed variables of Simpson, Pielou, and Shannon to the species diversity were 0.82, 0.23, and 0.799 (**Figure 4**), respectively. The path coefficients of understory herbaceous diversity stand structure and soil nutrient content were 0.35 and 0.23, respectively. The path coefficients between stand structure and soil nutrient content were 0.21, it was determined that alterations in the stand structure can also result in modifications to soil characteristics, which indirectly affect the diversity of understory herbaceous species.

The SEM was modified from the MI calculated using R. After modification, the values of CFI, AGFI, NFI, IFI, and PGFI were 0.950, 0.916, 0.939, 0.963, and 0.690, respectively, while the value of RMSEA was 0.053 (**Table 6**) (Zhao et al., 2023). Therefore, all fit indexes had an excellent fit for the requirements of fit reference values and the degree of adaptation (**Table 6**).

**Table 6:** Modified model fitness metrics for assessing the degree of adaptation for each indicator.

Model Fitting Index	Statistical Value	Reference Value	Degree of Adaptation
CFI	0.950	>0.9	Fit
AGFI	0.916	>0.9	Fit
NFI	0.939	>0.9	Fit
IFI	0.963	>0.9	Fit
PGFI	0.690	>0.5	Fit
RMSEA	0.053	<0.08	Fit

## 4 Discussion

### 4.1 Stand structure affects herbaceous plants species diversity

Stand structure reflects the process by which forest stands evolve and includes stand factors like stand density, mean DBH diameter, and variation in DBH diameter, which largely determine the species diversity of the understory vegetation (Tatsumi and Owari, 2013). Stand density is an important factor affecting the composition and distribution of understory plant species through its direct effect on soil physical and chemical properties, and its indirect effect on light penetration by altering canopy cover (Lin, 2013; Chen et al., 2020). Diameter at breast height (DBH) and stand species richness have a strong relationship with herbaceous vegetation conditions, which is essential to maintaining community biodiversity (Li et al., 2011). The spatial structure of the forest stand is closely related to each tree, which can reflect the size dominance difference between trees, horizontal spatial position distribution, and species complexity (Tanioka et al., 2022). Understory herbaceous plants are the primary contributors to the stand structure of forest ecosystems (Wei, 2020). The complexity of a multi-species community’s stand structure directly correlates with the availability and heterogeneity of the understory light environment, as well as the diversity of microhabitats (Mestre et al., 2017). Spatial heterogeneity is a crucial factor for the renewal of ecological niche differentiation. It also results in spatial variation in community species composition and diversity (Choe et al., 2021). The higher the overall biodiversity of the forest stand, the more pronounced these effects become (Ádám et al., 2013).

The study concluded from the univariate correlation analysis, typical correlation analysis, and multiple linear regression that the canopy density and tree density of the stand non-spatial structure were significantly and negatively correlated with understory herbaceous plant species diversity. The results of the study are consistent with Cui’s research (Cui et al., 2022). Pearson correlation analysis of the uniform angle, neighborhood comparison, and mingling degree were all spatial structure parameters on the horizontal plane, among which the neighborhood comparison had a significant effect on understory herbaceous plant species diversity (p< 0.05). The uniform angle reflected the horizontal distribution pattern of the stand. The stand distribution was random when the uniform angle was small, and it was aggregated when the uniform angle was large, and the effect of the mingling degree on understory herbaceous plant species diversity was reflected in the competition among trees, leading to mutual suppression between trees and the release of nutrient space for understory vegetation (Ali et al., 2016; Yang et al., 2023). The factors of canopy density and Hegyi competition index had large correlation coefficients with the linear combination V1 (species diversity variable group), of 0.32 and −0.201, respectively, indicating that the combined effect of canopy density and Hegyi competition index on understory herbaceous plant species diversity was the largest.

Canopy density, *N*, *M*_c,_ and *CI* played a key role in significantly affecting understory herbaceous plants species diversity from the multiple regression equations, understory vegetation can receive less light when the canopy density is high; therefore, the species diversity is limited by light conditions. In the linear combination, the loads of the Hegyi competition index and Canopy density were 0.872 and-0.506, respectively indicating that tree density was negatively correlated with species diversity in the herbaceous layer. The SEM combines path analysis Simpson and Shannon had the greatest weight among the herbaceous plants’ species diversity were 0.82 and 0.79, respectively; *N* and *HT* had the greatest weight among non-spatial structure indicators were -0.47 and 0.43, respectively; *M*_C_ and U had the greatest weight among spatial structure indicators were 0.42 and 0.41, respectively; and the path coefficients for the stand structure for the species diversity was 0.35 in our study. The results identified significant effects of different stand structures on herbaceous plants’ species diversity, with *M_c_*, *W*, *CI*, *N*, and Canopy density having the greatest effect among the stand structure indicators.

### 4.2 Nutrient contents of soils affect herbaceous plants species diversity

Soil factors can have a direct effect on the growth and distribution of understory herbaceous vegetation, which in turn affects the herbaceous plants’ species diversity (Meng et al., 2023). Soil organic carbon content can reflect soil fertility and directly influence the availability of nitrogen and phosphorus required for plant growth, which in turn affects plant growth and distribution, as well as the diversity index of the plant community (Schoenholtz et al., 2000).

Our results show that Pearson correlation analysis of the SOM, TP, TN, and AK had a significant effect on understory herbaceous plant species diversity (p< 0.05). Soil phosphorus and potassium levels depend on source material, degree of weathering, and leaching. The factors of SOM and TN had large correlation coefficients with the linear combination V1 (species diversity variable group), of 0.209 and 0.273, respectively, indicating that the combined effect of SOM and TN on understory herbaceous plants species diversity was the largest.

Soil is essential for the growth of vegetation, and soil moisture and nutrient levels are crucial for the growth, development, and diversity of plants (Kooch et al., 2017; De Groote et al., 2018). Studies have shown that plant diversity is positively correlated with soil organic carbon and total nitrogen. This conclusion is consistent with the findings of previous studies. This paper concludes, through multiple stepwise regression analyses, that SOM, TN, and AK have a contributing effect on the diversity index. The path coefficients for the observed variables including SOM, TN, and TP to total nutrient indicators were 0.86, 0.64, and 0.80 (**Figure 4**), respectively, and the path coefficients for the nutrient contents of soils for the species diversity were 0.23 in our study.

Soil factors play a pivotal role in determining the diversity of understory herbaceous species. They exert a direct influence on both the growth and distribution of understory herbaceous vegetation (Kaufmann et al., 2017; Wang et al., 2024). The results of this study indicate that soil organic matter, total nitrogen, and quick-acting potassium are the most significant contributors to the diversity of understory herbaceous species.

### 4.3 Stand structure indirectly affect herbaceous plants species diversity

Furthermore, it was determined that alterations in the stand structure (spatial structure and non-spatial structure) can also result in modifications to soil characteristics, which indirectly affect the diversity of understory herbaceous species (Alem et al., 2015).

The stand structure has the potential to influence the microenvironment and distribution pattern of understory herbaceous plants (Alaina et al., 2000). On the one hand, mixed forests comprising multiple species can serve to reduce the extent of ecological competition between species, thereby limiting competition between trees and providing favorable conditions for the growth of understory herbaceous plants (Cao et al., 2020). Conversely, the distribution of trees can directly influence the dimensions and positioning of the forest gap, regulating the intensity of effective light within the forest., and the redistribution of light, heat, water, and fertilizer within the forest to a certain extent affects the species and number of understory herbaceous plants and their distribution, particularly for light-loving species, the growth and development of these species are influenced by changes in light intensity, which indirectly affects the understory herbaceous plant species diversity (Lei et al., 2003; Zhang et al., 2022). The stand spatial structure has a great influence on soil nutrients, and it is attributable to the overlapping of the canopy in mixed forests comprising multiple species, which are prone to producing a greater quantity of litter, accelerating the decomposition of deadwood and increasing the likelihood of accumulating a substantial amount of organic matter (Cui et al., 2004; Zhang et al., 2022). Additionally, the root systems of mixed forests are complex and intertwined within the soil, which significantly enhances soil aeration, intensifies soil microbial activity, and facilitates soil nutrient cycling and utilization. This, in turn, markedly stimulates vegetation growth. Our results show that the path coefficient between stand structure and soil nutrient content was 0.21 following structural equation analysis, which indirectly affects the diversity of understory herbaceous species.

## 5 Conclusions

The stand structure had a direct and indirect effect on the sex of understory herbaceous species. The factors that had the greatest effect on the diversity of understory herbaceous species were *M_c_*, *W*, *CI*, *N*, and canopy density. Soil factors also had a significant effect on the diversity of understory herbaceous species. The factors that had the greatest effect on the diversity of understory herbaceous species were soil organic matter, total nitrogen, and quick-acting potassium. The path coefficients of understory herbaceous diversity stand structure and soil nutrient content were 0.35 and 0.23, respectively. In summary, both soil factors and stand spatial structure had a pronounced effect on the diversity of understory herbaceous species. However, the effect of stand structure on this diversity was greater than that of soil factor. In terms of mediating effects, alterations in stand structure will indirectly impact the diversity of understory herbaceous species due to their dependence on soil variables, as the aerosol factor is challenging to regulate by human means. However, modifications to the stand structure are a pivotal approach to enhancing the diversity of understory herbaceous species. This approach involves reducing both the density of the upper forest canopy and the density of trees. Additionally, it entails adjusting the extent of tree mixing and spatial distribution, which contributes to the overall stability of the diversity of the understory herbaceous species. There has been a net increase in overall diversity levels.

## Data Availability

The original contributions presented in the study are included in the article/supplementary material. Further inquiries can be directed to the corresponding author.
